# COVID-19 Emergency Management: From the Reorganization of the Endoscopy Service to the Verification of the Reprocessing Efficacy

**DOI:** 10.3390/ijerph17218142

**Published:** 2020-11-04

**Authors:** Beatrice Casini, Benedetta Tuvo, Fabrizio Maggi, Giuliana Del Magro, Alessandro Ribechini, Anna Laura Costa, Michele Totaro, Angelo Baggiani, Giulia Gemignani, Gaetano Privitera

**Affiliations:** 1 Department of Translational Research, N.T.M.S., University of Pisa, 56126 Pisa, Italy; tuvobenedetta@hotmail.it (B.T.); fabrizio.maggi@unipi.it (F.M.); anna.costa@med.unipi.it (A.L.C.); michele.totaro.unipi@hotmail.com (M.T.); angelo.baggiani@med.unipi.it (A.B.); gaetano.privitera@med.unipi.it (G.P.); 2 Virology Division, University Hospital of Pisa, 56126 Pisa, Italy; 3 Endoscopy Service Division, University Hospital of Pisa, 56126 Pisa, Italy; g.delmagro@ao-pisa.toscana.it; 4 Thoracic Endoscopy Division, University Hospital of Pisa, 56126 Pisa, Italy; a.ribechini@ao-pisa.toscana.it; 5 Medical Direction, University Hospital of Pisa, 56126 Pisa, Italy; g.gemignani@ao-pisa.toscana.it

**Keywords:** SARS-CoV-2, endoscopy, reprocessing, virological investigation, COVID-19

## Abstract

Microbiological surveillance carried out in order to verify the effectiveness of endoscope reprocessing does not include the research of viruses, although endoscopes may be associated with the transmission of viral infections. This paper reports the experience of the University Hospital of Pisa in managing the risk from severe acute respiratory syndrome coronavirus 2 (SARS-CoV-2) during an endoscopy. A review of the reprocessing procedure was conducted to assess whether improvement actions were needed. To verify the reprocessing efficacy, a virological analysis was conducted both before and after the procedure. Five bronchoscopes and 11 digestive endoscopes (6 gastroscopes and 5 colonoscopes) were sampled. The liquid samples were subjected to concentration through the use of the Macrosep Advance Centrifugal Devices (PALL Life Sciences, Port Washington, NY, USA) and subsequently analyzed using the cobas^®^ SARS-CoV-2 Test (Roche Diagnostics, Basel, Switzerland), together with eSwab 490 CE COPAN swabs (COPAN, Brescia, Italy), which were used to sample surfaces. In accordance with the first ordinance regarding the coronavirus disease 2019 (COVID-19) emergency issued by the Tuscany Region in March 2020, a procedure dedicated to the management of the COVID-19 emergency in endoscopic practices was prepared, including the reprocessing of endoscopes. The virological analysis carried out on samples collected from endoscopes after reprocessing gave negative results, as well as on samples collected on the endoscopy column surfaces and the two washer-disinfectors that were dedicated to COVID-19 patients. The improvement in endoscope reprocessing implemented during the COVID-19 emergency was effective in ensuring the absence of SARS-CoV-2, thus reducing the risk of infections after an endoscopy on COVID-19 patients.

## 1. Introduction

The risk of exposure and subsequent infection by severe acute respiratory syndrome coronavirus 2 (SARS-CoV-2) in healthcare professionals in endoscopy units has been recognized as potentially high since endoscopies, especially bronchoscopies, are classified as aerosol-generating procedures (AGPs). Infection can occur via inhalation of airborne droplets, conjunctival contact, and contact with surfaces in the immediate environment or with medical devices used on the infected patient (indirect contact) [1,2,3]. When patients with suspected or confirmed COVID-19 are undergoing a bronchoscopy, the use of appropriate personal protective equipment (PPE) by healthcare professionals was strongly recommended [3], although there are still discordant opinions on whether to perform this procedure, even if necessary, for the diagnosis of a malignant neoplasm. The stratification of patients undergoing endoscopic investigation for preventing infection in healthcare workers was proposed through a risk assessment based on symptoms and contact tracing. The recommendation to postpone the elective procedure was given for patients with suspected or confirmed COVID-19, but the management of asymptomatic patients who have positive screening results remains an open problem. However, screening in pre-hospitalization or in any case before the endoscopic procedure was recommended to identify positive patients, even in the asymptomatic phase, in order to implement the appropriate infection control procedures. The importance of asymptomatic patients in the SARS-CoV-2 transmission was underlined by several reports [4,5].

Regarding after the procedure, there is consensus on the importance of implementing a disinfection procedure on frequently touched surfaces, including those of electromedical devices, such as the monitor, and of verifying the correct execution of the reprocessing of reusable endoscopes [6,7].

The risk of SARS-CoV-2 infection is not limited to thoracic endoscopy; Perisetti et al. pointed out how this risk may also be related to digestive endoscopy due to the tropism of this virus for intestinal mucosal cells expressing the angiotensin-converting enzyme II receptor (ACE2) [8]. Although the viral loads in stool samples are lower than those of respiratory samples, values up to 1.21 × 10^5^ copies/mL were detected in patients without diarrhea and until 11 days after symptom remission, suggesting that there is a risk of virus transmission, even during endoscopy in asymptomatic patients [9]. Repici et al. reported that endoscopes used for the digestive tract can become contaminated, not only due to contact with fecal material but also during the biopsy procedure, stressing that adherence to endoscope reprocessing procedures is essential to reduce the risk [10].

The potential airborne transmission of enteric microorganisms during digestive endoscopy was recently demonstrated by Johnston et al. [11] after analyzing endoscopist doctors’ face shields: 45.8% (104/227) of the face shields were contaminated, 21.4% of which were when the operator was located at a distance of about 2 m. This distance allows for the diffusion of droplets, through which the transmission of SARS-CoV-2 takes place [12].

Although endoscopic gastrointestinal (GI) procedures may have different levels of risk, the same personal protective measures were recommended in the Position Statement of the European Society of Gastrointestinal Endoscopy and the European Society of Gastroenterology Nurses and Associates (ESGE/ESGENA) for all procedures in both the upper and lower tract. According to the patient’s risk status, PPE for low-risk patients includes gloves, a hair net, goggles (goggles or visor), a waterproof gown, booties/shoe covers, and a respiratory protective device (surgical mask). For high-risk or infected patients, in addition to the use of the PPE listed above, a high-filter respiratory mask (FFP2/3) and two pairs of gloves were also used [1]. Furthermore, the use of disinfectants with proven virucidal activity according to the EN 14885:2019 [13] standard was recommended, both for the reprocessing of the endoscopes and the disinfection of surfaces [1].

Ofstead et al. recently reported the transmission of secondary infections related to the use of bronchoscopes in patients with a SARS-CoV-2 infection [14]. In particular, pathogens with a water origin, such as *Stenotrophomonas* spp., *Pseudomonas* spp., and *Serratia* spp., were frequently isolated from bronchoalveolar lavages (BALs) of COVID-19 patients and from contaminated bronchoscopes [15]. The authors pointed out that high-level disinfection can theoretically eliminate this risk if the bronchoscopes are well maintained and reconditioned according to manufacturer’s instructions and recommendations. However, pathogens and, in particular, multidrug-resistant outbreak organisms were found in endoscopes due to errors in the execution of the reconditioning procedure or due to the presence of microlesions inside the endoscope channels that favored biofilm formation, facilitating microorganisms survival [16]. 

Due to the frequent occurrence of bronchoscope-related infections and the risk of SARS-CoV-2 infection for the healthcare personnel reconditioning this device, the use of disposable bronchoscopes, when available, was suggested as good practice [7].

Given these recommendations, this paper describes the experience of a teaching University Hospital in Central Italy regarding SARS-CoV-2 risk management in endoscopy, with a particular focus on the improvement of the reprocessing protocol in order to highlight improvement actions and guarantee safe processes.

## 2. Materials and Methods

### 2.1. Study Setting

The University Hospital of Pisa is a highly specialized teaching tertiary hospital with 1082 beds and was designated as one of the three referral hospitals for COVID-19 patients in the Tuscany Region in Italy. After the first Tuscany COVID-19 case, the hospital organized a multidisciplinary task force, which was composed of experts in infection prevention and control, occupational medicine, and hospital management. Based on the scientific data published since the start of the outbreak and on previous experience from former outbreaks (SARS and MERS), the task force developed a technical procedure to face the epidemic, which included the management of endoscopies.

**Figure 1:**
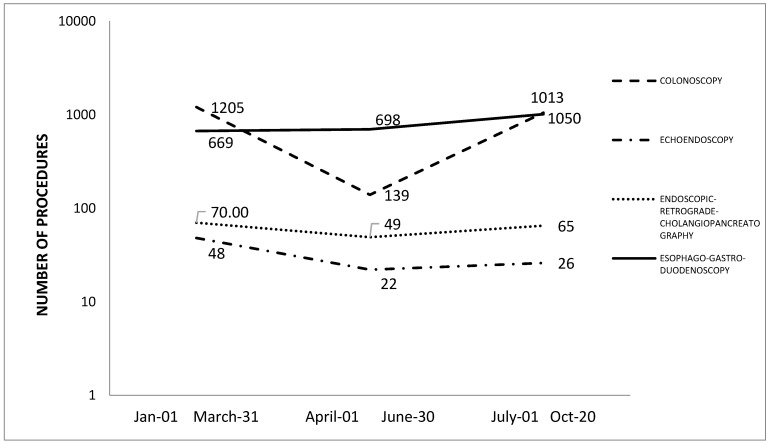
Number and type of endoscopy performed from January to October 2020.

In the Digestive Endoscopy Unit of the Hospital, about 13,000 endoscopic procedures are performed every year. In 2019, 13,222 colonoscopies, 14,735 gastroscopies, and 1100 endoscopic retrograde cholangiopancreatographies (ERCP) and ecoendoscopies in total were performed. During the pandemic, digestive endoscopic activity underwent a major decline, particularly in ordinary activity, which was largely related to cancer screening tests, as shown in Figure 1.

Following the declaration of the COVID-19 pandemic, the first ordinance on the COVID-19 emergency issued by the Tuscany Region in March 2020 suspended planned activities, both outpatient and surgical, leaving only the emergency pathways of class A oncology and other pathologies that cannot be postponed, including those carried out on COVID-19 patients through the activation of a dedicated care path.

In accordance with this ordinance, a procedure dedicated to the management of the COVID-19 emergency in endoscopic practices was prepared.

### 2.2. Evaluation of the Effectiveness of the Reprocessing Process

The reprocessing of flexible endoscopes and accessories was performed according to published guidelines [17] by using products with virucidal activity that were certified according to the EN 14476:2019 standard [18]. Briefly, for the pre-cleaning phase conducted at the patient’s bed immediately after the procedure, 250 mL of detergent/disinfectant solution (Neo Proteozim Plus 500, Cantel, Pomezia, RM, Italy) was sucked for 20 s through the suction channel of the endoscope attached from the light source. After running the leak test, manual cleaning was performed in accordance with the Instruction for Use of the manufacturer, using only single-use cleaning solutions, brushes, and other cleaning devices (sponges). Endoscopes were fully immersed in Neo Proteozim Plus 500 solution (Cantel Medical S.r.l, Pomezia (RM), Italy) at the concentration and for the contact times recommended (dilution 1:500 in drinking water, 10 min contact time). Finally, the endoscopes were disinfected in an automatic washer-disinfector (Soluscope series 4, Soluscope, Aubagne, France), complying with ISO 15883-1 and ISO 15883-4 [19,20], which involved applying the intensive high disinfection cycle that involved double washing with enzymatic detergent (Soluscope CLN, Aubagne, France) and high disinfection with peracetic acid at 900 ppm for 3 min at 40 °C (Soluscope PAA, Aubagne, France).

In order to verify the effectiveness of the reprocessing process, bronchoscopes and digestive endoscopes were selected and sampled after cleaning and disinfection. The inclusion criteria were as follows: endoscopes used on hospitalized patients with COVID-19 symptoms (dyspnoea, fever, myasthenia, gastrointestinal symptoms, dry cough, ageusia, or anosmia), a SARS-CoV-2-positive reverse transcription polymerase chain reaction (RT-PCR) test on a nasopharyngeal swab and/or broncho-alveolar lavage or rectal swab in the last two days prior to performing the procedure and/or bilateral distribution of ground-glass opacities revealed using a chest computed tomography (CT) [21]. The traceability of endoscopes was managed at the Hospital of Pisa thanks to the use of IT Soluscope software 2.0 (Soluscope, Aubagne, France); therefore, it was possible to trace the patient on whom the endoscope was used.

To verify whether the endoscopes were contaminated with SARS-CoV-2 prior to reprocessing, an Olympus BF1T180 bronchoscope and an Olympus GIF800 gastroscope (Olympus Italia s.r.l, Segrate (MI), Italy) were sampled after the use on hospitalized patients with COVID-19 symptoms. In particular, the bronchoscope was used on a patient hospitalized for 12 days with a diagnosis of severe acute respiratory syndrome due to a SARS-CoV-2 infection, as confirmed by chest CT and a positive SARS-CoV-2 RT-PCR test on a nasopharyngeal swab upon admission (cycle threshold (Ct) value of 26).

The gastroscope was used for an esophagogastric duodenoscopy on a patient hospitalized for 23 days with symptoms attributable to COVID-19 and confirmed using an RT-PCR test on a nasopharyngeal swab upon admission and subsequently 48 h before the endoscopy (Ct values of 23.5 and 28, respectively).

The endoscopy columns dedicated to the endoscopes used on COVID-19 patients and dedicated washer-disinfectors were sampled to verify the effectiveness of the disinfection on their surfaces.

The sampling of the endoscopes was carried out in accordance with the protocol reported in the document “Duodenoscope Surveillance Sampling and Culturing Protocols” [22], using eSwab 490 CE with liquid Amies preservation medium (COPAN Italia S.p.a, Brescia, Italy) for sampling the outer surface and valve port of the biopsy channel; urethral swabs (APTACA S.p.a, Cannelli, Italy) moistened with sterile phosphate buffer solution (VWR Chemicals, Radnor, Pennsylvania, United States) were used for sampling the internal channel of the distal end of the endoscope. As recommended by the CDC (Centers for Disease Control and Prevention) guideline, after swabbing, the urethral cottoned swab portion was cut off and placed in the sample container. The same solution was used for irrigating the internal channels, applying the flush–brush–flush method, then collecting it in the same sample container, as described in the guideline.

To test the washer-disinfector machine, eSwab 490 CE COPAN swabs (COPAN Italia S.p.a, Brescia, Italy) were used for samples collected from the internal surfaces of the tank, the sprinkling nozzles, and the internal channels of the endoscope’s connectors. The same type of swab was used to sample 10 × 10 cm surfaces of the endoscopy column, in particular, in the area of the connection for the cold light source that was not covered by the protective sheath.

### 2.3. Virological Analysis

Immediately after sampling, the swabs and the sample container were transported at a controlled temperature (4 ± 2 °C) to the laboratory of the hospital Virology Unit for the qualitative detection of the SAR-CoV-2 genome and other *Betacoronaviruses* of the *Sarbecovirus* subgenus, using the real-time RT-PCR protocol proposed by Corman et al. [23].

Before the molecular investigation, the liquid samples were subjected to concentration through the use of the Macrosep Advance Centrifugal Devices (30KD) (PALL Life Sciences, Port Washington, NY, USA) and subsequently analyzed using the cobas^®^ SARS-CoV-2 Test (Roche Diagnostics, Basel, Switzerland).

The automatic Roche cobas^®^ 6800/8800 System was used with the cobas^®^ SARS-CoV-2 Test (Roche Molecular Systems, Branchburg, NJ, USA). This test is a single-well dual-target assay, which includes both the specific detection of SARS-CoV-2 (which causes COVID-19) and pan-*Sarbecovirus* detection for the *Sarbecovirus* subgenus family that includes SARS-CoV-2. A 0.6 mL aliquot of each sample was added to a master mix containing an RNA internal control that was simultaneously extracted with the sample (non-*Sarbecovirus*-related RNA), a positive external control (plasmid DNA containing SARS-Cov-2 or pan*-Sarbecovirus* sequences), and a negative external control (poly(rA) RNA). Automated data management was performed by the manufacturer’s software (cobas 6800/8800 software), which assigned test results for all the tests. According to the manufacturer’s instructions, a tested sample was considered SARS-CoV-2 positive if the cobas^®^ test showed positive results, both for SARS-CoV-2 and pan-*Sarbecovirus* target genes or for the SARS-CoV-2 gene only. In the case of positivity for the pan-*Sarbecovirus* target gene, the result should be reported as SARS-CoV-2 presumptive positive. In particular, the Ct values reported as “detected” was Ct < 33.5 for SARS-CoV-2 and Ct < 36.4 for the pan-*Sarbecovirus* target gene. “Presumptive positive” instead corresponded to SARS-CoV-2 not detected (Ct > 33.5) and the pan-*Sarbecovirus* target gene being detected (Ct < 38). The instrument software version used was 01.03.08.1011 and the results were reviewed directly on the system screen and printed as a report.

## 3. Results

### How the Endoscopy Service Was Reorganized during the COVID-19 Pandemic

Before performing any type of endoluminal procedure, a general assessment of the urgency and need of the procedure was assessed. The healthcare workers were trained on the correct use of protective personal equipment (PPE), on donning and doffing procedures, and on the ways to access the dedicated premises where the endoscopy was performed. A dedicated disposable kit was set up (headgear, face shield, long-sleeved water-resistant gown or medical protecting coverall, double long nitrile gloves, waterproof leg covers, and class 2 or 3 filtering face-piece respirators FFP2 or FFP3).

The endoscopy procedures were carried out at the patient’s bed in the inpatient areas, intensive care units, and operating rooms identified as “COVID-19 areas”. Special measures were taken, such as lowering the atmospheric pressure, where possible. In the operating room, endoscopies were often performed on patients in which pathologies of the digestive tract were found, such as perforation or obstruction of the esophagus. Patients from the emergency room had direct access to the endoscopy service only if the result of molecular screening for SARS-CoV-2 was negative and/or they required procedures that could not be postponed; in the event of an ongoing outcome, the patient was considered as possibly COVID-19 positive and the same protective measures were applied as for a confirmed COVID-19 patient. In this case, the patient accessed the service following a telephone agreement with the healthcare workers such that upon their arrival, the dedicated staff of the reception carried out the COVID-19 pre-endoscopy triage and could guarantee that the dedicated outpatient area was available. All patients were questioned about contact with COVID-19-positive individuals and recent or present symptoms, such as fever, cough and dyspnoea, rhinitis, and sudden loss of smell and/or taste. Before entering the waiting room, the patient had to wear a surgical mask and gloves and undergo a temperature measurement and rapid molecular testing to determine whether COVID-19 was present. As recommended by ESGE/ESGENA [1], risk stratification of the patients for possible COVID-19 infection should be done 1 day prior to the GI endoscopy and then again on the day of the endoscopy by questioning for symptoms and contacts, or through tests for virus infection or immunity. Although many of the COVID-19 symptoms are similar to those of other respiratory diseases, fever (body temperature > 37.5° or 38 °C) is one of the quite frequent symptoms of COVID-19 and the temperature check on arrival in the unit may help to identify COVID-19 patients. To test for virus infection, the nasopharyngeal swab for viral genome detection remains the gold standard for diagnosing COVID-19 [24].

In the case of a patient that cannot be transported to the emergency room, the healthcare workers (one doctor and two nurses) were activated to carry out the endoscopy in the dedicated operating room. In the COVID-19 area, each workstation for endoscopy was equipped with dedicated single and reusable material, from the endoscopy column to the endoscopes, up to the containers for the safe transport of the contaminated device.

The Thoracic Endoscopy Unit and intensive care units were equipped with disposable bronchoscopes for bronchial inspection and for performing BAL when necessary.

Disinfection of the endoscopy room surfaces was performed after each procedure using a disinfectant that is effective against viruses. In this hospital, housekeeping staff applied a chlorine-based disinfectant (0.5% sodium hypochlorite, 5000 mg/L) with a 10 min contact time for the disinfection of compatible surfaces [25], whereas on electromedical non-invasive devices, ready to use wipes pre-moistened with 70% isopropyl alcohol were used with 15 s of contact time.

Regarding the reprocessing of endoscopes, the analysis of the procedure identified critical issues for which improvement actions were taken to reduce the risk of transmitting SARS-CoV-2 infection. The interventions concerned:

1. The identification of endoscopes (colonoscopes, gastroscopes, and bronchoscopes), endoscopy columns, and electrosurgical units dedicated for use on COVID-19 patients. Columns were covered by a disposable sheath and at the end of each procedure, the column was subjected to sanitization in accordance with the manufacturer’s instructions. For the disinfection of compatible surfaces, 0.5% sodium hypochlorite, prepared at the moment, was used, while for the monitor, disposable wipes impregnated with 70% isopropyl alcohol were used. Wipes were chosen on the basis of their compliance with the EN 14476:2019 standard [18]. Each column was equipped with a single-patient irrigation bottle and accessory tubing. The transport of the endoscopes in the reprocessing room was organized in dedicated containers and identified with an appropriate color code and risk identification. Care was taken to ensure that the exterior of the containers was not contaminated when loading.
2. For the phase involving pre-cleaning of the endoscope, which is conducted at the patient’s bed immediately after the procedure, and for the manual cleaning phase performed in the reprocessing room, the use of enzymatic detergent with disinfectant activity was recommended. The certification of reduction of the viral load according to the EN 14476:2019 standard was required in order to reduce the level of the endoscope contamination, and consequently, to prevent the formation of infectious aerosols, in particular, in the irrigation and brushing of the internal channels phases [1,10]. Disposable valves were already in use before the COVID-19 emergency. The staff were equipped with the appropriate PPE, which was set up inside the dedicated disposable kit. Due to the limited availability of PPE, face shields were disinfected after use by applying 0.5% sodium hypochlorite, prepared at the moment. In the cleaning phase, the automatic irrigation pump was not recommended in order to avoid contamination since the endoscopy unit did not yet have sterilizable fittings for endoscopes that were potentially contaminated with SARS-CoV-2.
3. The leak testing was not recommended in wet conditions due to the risk of aerosol generation. For this reason, dry leak testing was performed in the procedure room while staff were still wearing PPE. Confirmation of a leak test was obtained subsequently within the washer-disinfector cycle.
4. Two washer-disinfectors (Soluscope series 4, Soluscope, Aubagne, France) were dedicated to the high-disinfection phase. These machines comply with ISO 15883-1 and ISO 15883-4; therefore, they also guarantee efficacy (high disinfection) against viruses, in particular, on adenovirus type 5, poliovirus type 1, and bovine parvovirus Haden strain [19,20]. The manufacturer also provided the certification of virucidal activity according to the EN 14476+A1:2015 standard [18]. The intensive high-disinfection cycle was recommended for use. It involves double-washing with enzymatic detergent and high disinfection with peracetic acid at 900 ppm for 3 min at 40 °C. Regarding bronchoscopes, based on their compatibility, hydrogen peroxide gas plasma sterilization (59% H_2_O_2_, 55 min, 45 °C) was recommended.
5. For the storage phase, endoscope storage drying cabinets (DSC8000-Soluscope, Soluscope Aubagne, France) that conformed to ISO 16442:201519 were identified as suitable because they allowed for better drying of the internal channels of the endoscopes and were equipped with a UV-C system (254 nm), which guarantees the hygienic quality of the storage environment [26]. Specific drawers and connectors were identified for endoscopes that were dedicated to COVID-19 patients.

Finally, to ensure that the endoscope reprocessing procedures were carried out correctly, audits were conducted in the two endoscopy units.

Endoscopes that were used on SARS-CoV-2-positive patients who met the selection criteria and were hospitalized in the COVID-19 area, namely, 4 bronchoscopes and 10 digestive endoscopes (5 gastroscopes and 5 colonoscopes), were sampled within three days of reprocessing (Table 1). 

One column and two washer-disinfectors dedicated to COVID-19 patients were also sampled.

In contrast, before the reprocessing, the virological analysis carried out on the Olympus BF1T180 bronchoscope revealed the presence of SARS-CoV-2 RNA in the sampling liquid collected from the endoscope, with a Ct value of 30.5, while the eSwab COPAN swabs collected from the outer surface and valve port of the biopsy channel were negative.

On the Olympus GIF800 gastroscope, SARS-CoV-2 RNA was detected in the sampling liquid with a Ct value of 32. In this case, the surface swabs also gave negative results for the RT-PCR tests.

The virological analysis carried out on both the swabs and the liquids collected from endoscopes gave negative results on all the analyzed samples; therefore, no sample required isolation on cell cultures. Furthermore, no traces of SARS-Cov-2 RNA were found on samples collected on the endoscopy column surfaces or on the two washer-disinfectors dedicated to COVID-19 patients. None of the results obtained were classified as “invalid” based on the compliance of the amplification of controls.

Throughout COVID-19 pandemic phase one, no endoscopy healthcare workers were tested positive for SARS-COV-2.

## 4. Discussion

Endoscopy is an important procedure for diagnosing and staging cancer but the COVID-19 emergency has greatly reduced the possibility of performing it in symptomatic or patients classified as “at risk”.

Healthcare professionals in endoscopy units are at high risk of SARS-CoV-2 infection and although control measures, such as the use of appropriate PPE, have been shown to be effective in ensuring the safety of both healthcare workers and patients, during the COVID-19 pandemic, we had a significant reduction in the number of endoscopic procedures performed.

Postponing an endoscopy for 6 weeks has been estimated to represent a low risk for the great majority of patients awaiting a diagnosis [27], but most of the endoscopy units resumed ordinary activity after more than 4 months, as happened in the University Hospital of Pisa. Every effort made to counter the COVID-19 pandemic was useful for the resumption of ordinary healthcare activity.

Throughout the COVID-19 emergency, it is also important to guarantee the quality of the reprocessing of endoscopes since we do not yet have enough evidence regarding the survival of this virus outside the host and its resistance to disinfectants.

Microbiological surveillance that is carried out in order to verify the effectiveness of the reprocessing procedures does not include the search for viruses, although these devices can be associated with the transmission of viral infection during endoscopic activity.

Birnie et al. reported six cases of hepatitis B virus (HBV) infection by the same subtype (ay), which were related to endoscopy from an instrument sterilized by immersion in activated glutaraldehyde for 21 h (three times longer than the recommended period) and used on the previous day on a patient with bleeding oesophageal varices who was incubating type B viral hepatitis [28].

The virological investigation was based on protocols that are not yet standardized and not always able to detect viral infectivity. The detection of the viral genome using a polymerase chain reaction, for example, can allow for verifying the virus integrity only if photo-reactive DNA intercalating dyes, such as ethidio monoazide or propidium monoazide, are used. These dyes prevent the amplification of genomes outside the viral structure or of genomic fragments inside damaged viruses by binding the double helix of the genome and inhibiting the polymerase reaction [29]. This possibility is clearly not applicable to single-stranded viruses.

In this study, the presence of SARS-CoV-2 was studied on bronchoscopes and digestive endoscopes (five bronchoscopes, six gastroscopes, and five colonoscopes) used on patients diagnosed with COVID-19. To verify whether the endoscopes were contaminated with SARS-CoV-2 prior to reprocessing, two endoscopes (one bronchoscope and one gastroscope) were sampled after their use on hospitalized patients with COVID-19 symptoms and SARS-CoV-2 RNA was detected on both endoscopes. The sampling technique showed a good recovery of SARS-COV-2, which was thanks to the use of the Macrosep Advance Centrifugal Devices that allowed for concentrating the virus in the volume that was strictly required by the Roche cobas^®^ 6800 System analysis.

Our results showed that the reprocessing procedure, which was improved by implementing the described corrective actions, was effective at reducing the risk of SARS-CoV-2 infections associated with the use of endoscopes.

Our study had several limitations. The detection of RNA using RT-PCR-based assays is not necessarily indicative of a viable virus that could be transmissible and capable of causing infection. Our method did not include pre-treatment of the sample with DNA intercalating dyes; however, if we had had any positivity, we would have verified infectivity through cultivation on permissive cells. In our opinion, the RT-PCR analysis can be considered as an indicator of the endoscope disinfection effectiveness and as a proxy measurement of infectivity based on the general principle that residual traces of biological molecules on reprocessed devices represent a potential marker of sanitation failure [30]. Moreover, in our study, negative results were obtained by the cobas^®^ SARS-CoV-2 test when the fluorescence reached the Ct values of >33.5 for SARS-CoV-2 and >36.4 for the pan-*Sarbecovirus* target gene. Many qPCR assays use a Ct cutoff of 40, which allows for the detection of very few starting RNA molecules but we must take into account that it is not possible to compare Ct values obtained using other methods having different viral load kinetics.

This study also has strengths, as follows: it is the first study to demonstrate the absence of the SAR-CoV-2 genome on reprocessed endoscopes used on patients with a COVID-19 diagnosis. Moreover, the analysis was carried out on the entire aliquot of the liquid sample thanks to its concentration with the Macrosep Advance Centrifugal Devices (30KD), as requested by the CDC guidelines [3].

## 5. Conclusions

In these months of the COVID-19 emergency, the personnel of the endoscopy units have had to make a considerable effort to reorganize the emergency procedures and to reopen the ordinary endoscopic service. Improving knowledge on preventing the spread of this disease is important for providing a basis to work on.

In this study, the virological analysis carried out on endoscopes where the presence of SARS-CoV-2 was found allowed us to demonstrate that the improvement of the reprocessing process that aimed to reduce the risk of SARS-CoV-2 infections was effective in ensuring the absence of the viral genome and in reducing the risk of SARS-CoV-2 infections after endoscopy on COVID-19 patients.

Particular attention must be paid to the risk of SARS-CoV-2 survival on surfaces within the endoscopy room and during procedures such that the correct disinfection of environments can be established. The choice of effective sanitation procedures will have to evaluate both the efficacy against SARS-CoV-2 and the effects on the microbial ecosystem in order to limit the spread of antimicrobial resistance. [31]

With the resumption of the endoscopic activity of the colorectal screening that was planned in phase two and is to be completed in phase three of the pandemic, the management of this risk appears particularly urgent in order to guarantee the safety of patients and healthcare workers.

## Figures and Tables

**Table 1:** Sampling dates and results of the selected endoscopes.

Brand	Type of Endoscope and Model	Age	Date of Use	Date of Reprocessing	Date of Sampling	Sites of Sampling	SARS-CoV-2 RNA
Olympus	Gastroscope	13	24.04.20	24.04.20	24.04.20	eSwab COPAN 1	Negative
	GIF800					eSwab COPAN 2	Negative
						GIF800	
Olympus	Gastroscope	13	30.04.20	30.04.20	30.04.20	eSwab COPAN 1	Negative
	GIF800					eSwab COPAN 2	Negative
						Liquid	Negative
Pentax	Colonscope	8	23.04.20	23.04.20	24.04.20	eSwab COPAN 1	Negative
	EC878					eSwab COPAN 2	Negative
						Liquid	Negative
Olympus	Colonscope	15	24.04.20	24.04.20	27.04.20	eSwab COPAN 1	Negative
	CF BIC 094					eSwab COPAN 2	Negative
						Liquid	Negative
Olympus	Gastroscope	18	24.04.20	24.04.20	27.04.20	eSwab COPAN 1	Negative
	GIF BIC 238					eSwab COPAN 2	Negative
						Liquid	Negative
Pentax	Gastroscope	8	29.04.20	29.04.20	30.04.20	eSwab COPAN 1	Negative
	EG 040					eSwab COPAN 2	Negative
						Liquid	Negative
FujiFilm	Gastroscope	1	05.05.20	05.05.20	06.05.20	eSwab COPAN 1	Negative
	FG098					eSwab COPAN 2	Negative
						Liquid	Negative
Pentax	Colonscope	14	06.05.20	06.05.20	07.05.20	eSwab COPAN 1	Negative
	EC333					eSwab COPAN 2	Negative
						Liquid	Negative
Pentax	Colonscope	14	06.05.20	06.05.20	07.05.20	eSwab COPAN 1	Negative
	EC 306					eSwab COPAN 2	Negative
						Liquid	Negative
Olympus	Colonscope	5	21.05.20	21.05.20	22.05.20	eSwab COPAN 1	Negative
	GIF241					eSwab COPAN 2	Negative
						Liquid	Negative
Olympus	Broncoscope	11	24.04.20	24.04.20	27.04.20	eSwab COPAN 1	Negative
	BF1T180					eSwab COPAN 2	Negative
						Liquid	Negative
Olympus	Broncoscope	11	29.04.20	29.04.20	30.04.20	eSwab COPAN 1	Negative
	BF1T180					eSwab COPAN 2	Negative
						Liquid	Negative
Olympus	Broncoscope	11	05.05.20	05.05.20	05.05.20	eSwab COPAN 1	Negative
	BF1T180					eSwab COPAN 2	Negative
						Liquid	Negative
Olympus	Broncoscope	9	20.05.20	20.05.20	22.05.20	eSwab COPAN 1	Negative
	BF1T180					eSwab COPAN 2	Negative
						Liquid	Negative

Note: eSwab COPAN 1—external surface of endoscope; eSwab COPAN 2—valve port of the biopsy channel; Liquid—solution used for irrigation of the internal channels of the endoscope.
